# Volume histogram analysis of whole-lung CT: differentiating usual from nonspecific interstitial pneumonias and predicting prognosis

**DOI:** 10.1007/s11604-025-01880-9

**Published:** 2025-10-10

**Authors:** Tomonori Chikasue, Hiromitsu Sumikawa, Akiko Sumi, Kotaro Matsumoto, Kenta Murotani, Shuichi Tanoue, Toru Arai, Shigeki Shimizu, Yoshikazu Inoue, Takeshi Johkoh, Yoshiaki Zaizen, Masaki Okamoto, Masaki Tominaga, Kiminori Fujimoto

**Affiliations:** 1 Department of Radiology, Kurume University School of Medicine, 67 Asahi-Machi, Kurume, Fukuoka, 830-0011 Japan; 2 Department of Radiology, NHO Kinki Chuo Chest Medical Center, Sakai, Osaka Japan; 3 Department of Health Care Administration and Management, Graduate School of Medical Sciences, Kyushu University, Fukuoka, Fukuoka Japan; 4 School of Medical Technology and Biostatistics Center, Kurume University, Kurume, Fukuoka Japan; 5 Clinical Research Center, NHO Kinki Chuo Chest Medical Center, Sakai, Osaka Japan; 6 Department of Pathology, NHO Kinki Chuo Chest Medical Center, Sakai, Osaka Japan; 7 Department of Internal Medicine, Osaka Anti-Tuberculosis Association, Osaka Fukujuji Hospital, Neyagawa, Osaka Japan; 8 Department of Radiology, Kansai Rosai Hospital, Amagasaki, Hyogo Japan; 9 Division of Respirology, Neurology, and Rheumatology, Department of Medicine, Kurume University School of Medicine, Kurume, Fukuoka Japan; 10 Department of Respirology, NHO Kyushu Medical Center, Fukuoka, Fukuoka Japan

**Keywords:** Computer-aided diagnosis, Volume histogram analysis, Usual interstitial pneumonia, Nonspecific interstitial pneumonia, Prognosis

## Abstract

**Purpose:**

Low agreements among experts for differentiating usual interstitial pneumonia (UIP) from nonspecific interstitial pneumonia (NSIP) motivate the use of automated imaging diagnosis. Volume histogram analysis (VHA) of the lung parenchyma using computer-aided diagnostic software is more straightforward to perform and interpret than radiomics. To assess whether a predictive model generated by VHA (VHA model), using voxel data of each lung lobe obtained via whole-lung CT, can differentiate radiological UIP from NSIP, and to explore the relationship between VHA model outcomes and patient prognosis.

**Materials and methods:**

This study included 74 patients from one university hospital (cohort A: 47 patients with idiopathic pulmonary fibrosis [IPF]/UIP and 27 with idiopathic NSIP [iNSIP] and connective tissue disease-associated NSIP [CTD-NSIP]) and 146 patients from another hospital (cohort B: 111 with IPF/UIP and 35 with iNSIP/CTD-NSIP), with diagnoses confirmed through multidisciplinary discussion. Using the VHA values obtained from each lung lobe in cohort A, a formula-based VHA model was developed. The regularization parameters were optimized using five-fold cross-validation to maximize the area under the receiver operating characteristic curve (AUC). This VHA model was externally validated in cohort B. The correlation between various parameters and prognosis was analyzed using Cox proportional hazards multivariate analysis.

**Results:**

The mean AUC of the best VHA model that differentiated UIP patterns in cohort A was 0.91 (95% confidence interval [CI], 0.84–0.98), with a positive predictive value (PPV) of 0.97 (0.88–1.00). External validation of this model for cohort B revealed that the AUC for UIP differentiation was 0.81 (0.70–0.88), with a PPV of 0.94 (0.88–0.98). Multivariate analysis revealed that the values calculated by the VHA model were correlated with prognosis (hazard ratio, 1.60; 95% CI, 1.17–2.18; *p* = 0.003).

**Conclusion:**

The VHA model could effectively differentiate radiological UIP patterns and may help predict the prognosis of patients with interstitial pneumonia.

**Secondary abstract:**

A formula-based model using CT volume histogram analysis (VHA) of each lung lobe was developed to differentiate usual interstitial pneumonia (UIP) from nonspecific interstitial pneumonia (NSIP). The VHA model demonstrated strong diagnostic performance, achieving an area under the curve of 0.81 in external validation, and also statistically correlated with patient prognosis.

**Supplementary Information:**

The online version contains supplementary material available at 10.1007/s11604-025-01880-9.

## Introduction

The diagnosis and severity assessment of interstitial lung diseases (ILDs) and the determination of treatment strategies require a comprehensive assessment of clinical information and high-resolution computed tomography (CT) data, which are essential for evaluating the characteristics, localization, and associated findings of abnormal lung opacities. Treatment decisions for ILDs, particularly idiopathic pulmonary fibrosis (IPF), heavily rely on accurate diagnosis. Therefore, it is critical to identify specific patterns on high-resolution CT, such as honeycombing and traction bronchiectasis [1–3]. However, as ILDs have diverse imaging manifestations, the diagnostic quality is influenced by the observer’s experience and reproducibility of image interpretations. Studies have shown relatively low-to-moderate agreements regarding high-resolution CT patterns in diagnosing ILDs even among experts, with kappa values of 0.62–0.69 for traction bronchiectasis [4, 5] and 0.40–0.58 for honeycombing [6]. When multidisciplinary teams assessed cases of IPF and nonspecific interstitial pneumonia (NSIP), which are common types of interstitial pneumonia [7, 8], the agreement varied, with weighted kappa coefficient values of 0.71 and 0.42, respectively [9]. Such variability in expert evaluations highlights the need to enhance imaging diagnostic capabilities for interstitial pneumonia through imaging analysis of information technology and medical artificial intelligence data for automated imaging diagnosis [10–16].

Some tools available for lung CT analysis focus on characterizing and quantifying lung parenchymal findings [10, 11], whereas others utilize deep learning to analyze and map abnormal lung findings [12–15, 17, 18]. Volume histogram analysis (VHA) of three-dimensional data obtained via whole-lung CT is a classical method providing a statistical representation of the distribution of voxel attenuation values within the lung. VHA of the lung parenchyma including abnormal findings, using computer-aided diagnostic software is more straightforward to perform and interpret than radiomics, which involves large amounts of high-dimensional data and complex feature extraction processes. However, only a few reports have investigated the potential of VHA to classify interstitial pneumonia imaging patterns and conduct prognostic analysis [19–22]. Although these studies of lung image analysis have been performed on the entire lung or on individual transaxial sections, it would be possible to develop models by performing VHA on each lung lobe and then integrating them.

This study aimed to assess whether a predictive model generated by VHA (VHA model), using voxel data from each lung lobe obtained via whole-lung CT, can effectively differentiate radiological usual interstitial pneumonia (UIP) from NSIP patterns and to explore the relationship between VHA model outcomes and patient prognosis in interstitial pneumonia.

## Materials and methods

This retrospective study was approved by the Ethics Committee of Kurume University (approval number: 22055) and the Ethics Review Board of NHO Kinki Chuo Chest Medical Center (approval number: 2022-107), and informed consent was obtained through opt-out processes.

### Patients

Considering the updated international multidisciplinary classification of idiopathic interstitial pneumonias published in 2013 [23], this study included consecutive adult patients with UIP patterns of IPF and those with NSIP patterns of idiopathic NSIP (iNSIP) and connective tissue disease-associated NSIP (CTD-NSIP) who underwent multidisciplinary discussion (MDD) between April 2014 and March 2021 at two hospitals. The "UIP pattern" in this study consisted mainly of cases with definite UIP (with honeycombing) and a small number of cases of probable UIP based on the official ATS/ERS/JRS/ALAT clinical practice guideline published in 2018 [24], and the diagnosis of IPF/UIP was confirmed by MDD through central review at each hospital. The "NSIP pattern" in this study refers to the NSIP pattern on HRCT, as defined by international guidelines [23, 24] and determined by MDD, regardless of etiology. Diseases showing an NSIP pattern on imaging often include cases initially classified as idiopathic that later develop CTD. Therefore, distinguishing idiopathic from CTD-associated cases based solely on imaging findings is often difficult. Clinically, as many cases showing the NSIP pattern require anti-inflammatory and/or immunosuppressive therapy, it is critically important to distinguish these cases from IPF/UIP as part of a single disease spectrum. Consequently, this study selected cases showing the NSIP pattern based on this concept.

**Fig. 1:**
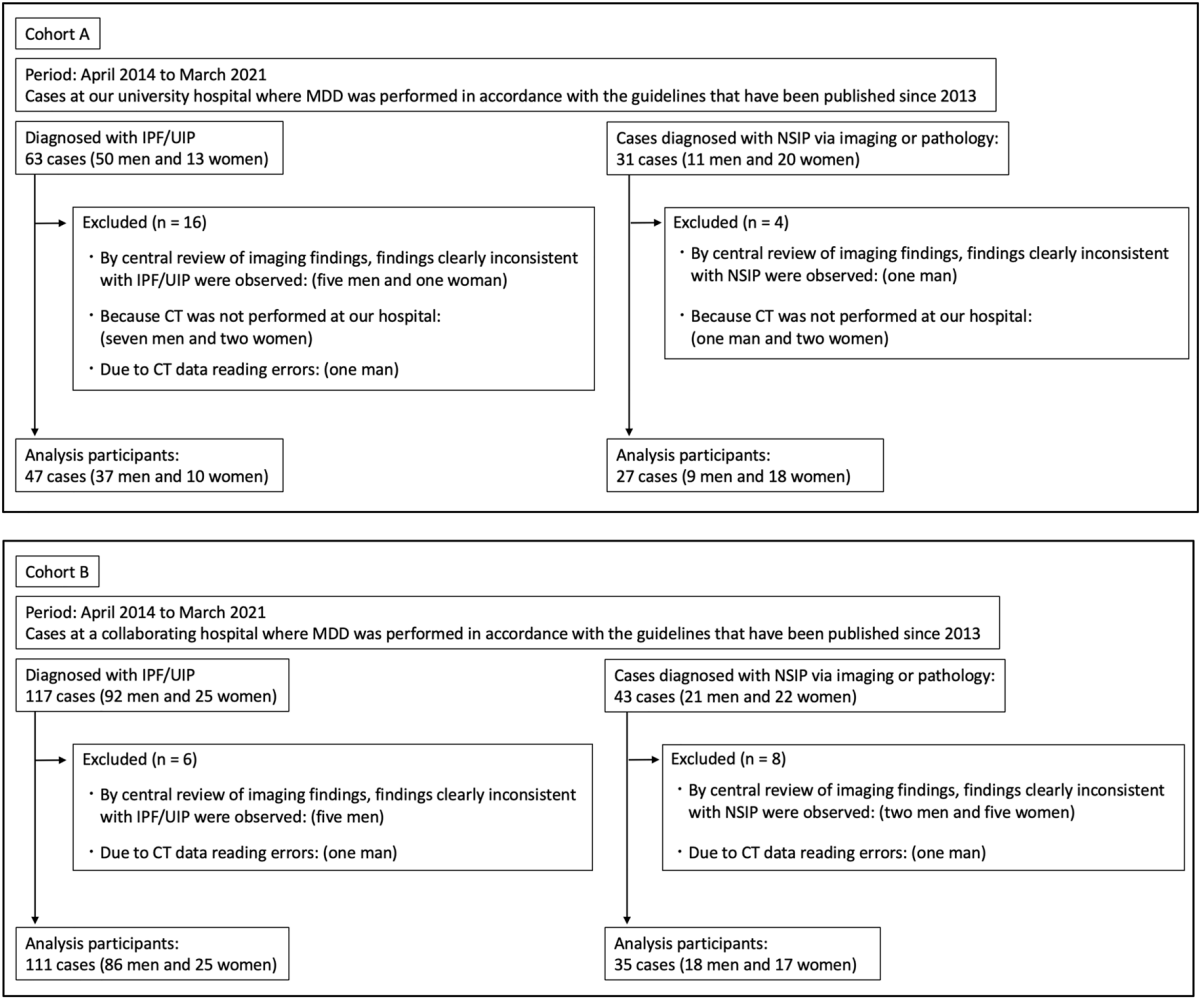
Flowchart of patient selection for cohorts A and B. **A** Flowchart showing final patient selection in cohort A. **B** Flowchart showing final patient selection in cohort B. MDD multidisciplinary discussion, IPF idiopathic pulmonary fibrosis, UIP usual interstitial pneumonia, NSIP nonspecific interstitial pneumonia, CT computed tomography

After applying the exclusion criteria, 47 patients with IPF/UIP (37 men, 10 women) and 27 with NSIP patterns (nine men, 18 women) were selected from Kurume University Hospital (cohort A), and 111 patients with IPF/UIP (86 men, 25 women) and 35 patients with NSIP patterns (18 men, 17 women) from NHO Kinki Chuo Chest Medical Center (cohort B). Figure 1 and Appendix S1 present the patient selection process. ILD diagnosis was confirmed through MDD among experienced clinical experts, radiologists, and pathologists based on the global guidelines [23, 24] and available high-resolution CT data. CTD diagnosis was made by expert rheumatologists. Details of the disease composition of the IPF/UIP and NSIP pattern groups in each cohort are provided in Appendix S2. Data on patient sex, age, smoking history (pack-years), cause-specific survival (CSS) and overall survival (OS), survival status, and follow-up periods were retrospectively obtained from medical records. Age and smoking history were the same as those at the time of the CT performed for VHA. Survival status was assessed between the day of interstitial pneumonia diagnosis and final observation day (August 31, 2023). For details of the “Whole-Lung CT Scans” and “Pulmonary Function Tests” conducted in this study, please refer to Appendix S3 and Appendix S4.

### VHA of the lungs

VHA of the lungs was performed using commercial software (SYNAPSE VINCENT®, Fujifilm Medical Co., Tokyo, Japan) [25–27]. The software automatically segmented each lung lobe (right upper, middle, and lower lobes; left upper and lower lobes). After visually confirming the absence of significant segmentation errors, further manual adjustments were not made. Then, we calculated kurtosis, skewness, and entropy for each lobe and for whole-lung (i.e., all five lobes combined). A representative example is shown in Figure S1.

### Development of the best model (i.e., the VHA model) for differentiating UIP from NSIP patterns

Clinical prediction models for differentiating UIP from NSIP patterns were developed via Least Absolute Shrinkage and Selection Operator (LASSO) using cohort A data as follows: model (a) based on only kurtosis data, model (b) only skewness data, model (c) only entropy data, and model (d) all three combined data. Model performance was optimized via five-fold cross-validation to maximize the area under the receiver operating characteristic curve (AUC), and each model was then validated in cohort B. The model with the highest AUC and best calibration plot was designated as the “VHA model.” We determined cutoff value using the Youden index and calculated sensitivity, specificity, accuracy, positive predictive value (PPV), and negative predictive value (NPV).

### Statistical analysis

Continuous variables were expressed as medians with the 25th–75th percentile of interquartile ranges (IQR). Comparisons of continuous variables were performed using nonparametric methods (Mann–Whitney U test or Kruskal–Wallis H test). The chi-square test was used for categorical variables. The 95% confidence intervals (CIs) for the evaluation index were calculated using the DeLong method.

The Kaplan–Meier survival curves of the OS and CSS for the groups diagnosed with IPF/UIP and NSIP patterns through MDD were compared with those classified into the IPF/UIP and NSIP pattern groups by the VHA model. The correlation between VHA model and prognosis was examined using Cox regression adjusted for age, sex, pulmonary function tests, smoking history (pack-years), and institutional diagnosis (IPF/UIP). Furthermore, Cox regression adjusted for the gender–age–physiology (GAP) score [28], smoking history, and institutional diagnosis was performed.

All statistical analyses were performed using R (version, 4.2.1) and IBM SPSS Statistics (version 29.0.2.0). The “glmnet” and “CalibrationCurves” packages in R were used for binary logistic regression using LASSO. *P* values < 0.05 were considered to indicate statistical significance. For the comparisons in Table 2, *p* values were adjusted using the Bonferroni correction for three families of metrics (kurtosis, skewness, and entropy). An adjusted *p* value < 0.05 was considered statistically significant.

## Results

### Patients

Table 1 summarizes the characteristics of each cohort. Significant differences were observed in several variables between the IPF/UIP and NSIP groups within each cohort. For cohort A, significant differences were noted in sex, age, smoking (pack-years), diffusing capacity of the lung for carbon monoxide (DLCO), percent DLCO (%DLCO), and GAP scores. For cohort B, significant differences were observed in sex, age, smoking (pack-years), percent predicted FVC (%FVC), DLCO, and GAP scores.

**Table 1:** Patient Characteristics

	Cohort A				Cohort B		
	IPF/UIP	NSIP Group	*P* value		IPF/UIP	NSIP Group	*P* value
Total no	47	27			111	35	
Sex (man/woman)	37/10	9/18	< 0.001		86/25	18/17	0.003
Age	70.0 (66.0–75.0)	61.0 (56.5–65.5)	< 0.001		72.0 (65.5–76.5)	68.0 (58.5–75.0)	0.026
Smoking (pack-years)	33.0 (0–58.8)	0.0 (0–17.8)	0.002		37.3 (15.8–56.8)	0.0 (0–26.5)	< 0.001
FVC	2.5 (1.9–3.1)	2.5 (2.0–3.1)	0.908		2.5 (1.8–3.2)	2.7 (1.9–3.4)	0.239
%FVC	78.9 (63.5–93.6)	86.2 (70.8–98.4)	0.115		83.7 (61.8–97.5)	95.4 (79.9–103.2)	0.018
FEV_1_	2.0 (1.6–2.5)	1.9 (1.5–2.3)	0.596		2.0 (1.5–2.5)	2.1 (1.5–2.8)	0.338
FEV_1_%	81.1 (76.7–87.1)	80.3 (74.1–83.8)	0.222		83.0 (78.7–86.7)	81.4 (77.5–85.3)	0.259
DLCO	10.2 (7.3–12.9)	12.9 (10.8–16.8)	0.005		10.6 (7.2–13.8)	12.7 (9.2–16.1)	0.015
%DLCO	63.2 (43.8–81.8)	74.0 (60.6–91.9)	0.026		63.0 (48.8–81.9)	73.0 (53.2–84.9)	0.218
GAP score	3.0 (3.0–4.0)	0.0 (− 1.0 to 0.0)	< 0.001		3.0 (2.0–4.0)	1.0 (0.0–1.0)	< 0.001

Values are expressed as median (25th–75th percentile of the interquartile range). *P* values were calculated using the χ2 test for sex differences and the Mann–Whitney U test for other variables. The NSIP group consisted of patients with idiopathic nonspecific interstitial pneumonia (iNSIP) and connective tissue disease-associated nonspecific interstitial pneumonia (CTD-NSIP)

IPF idiopathic pulmonary fibrosis, UIP usual interstitial pneumonia, FVC forced vital capacity, %FVC percent predicted FVC, FEV1 forced expiratory volume in one second, FEV1% FEV1 percent, DLCO diffusing capacity of the lung for carbon monoxide, %DLCO percent DLCO, GAP gender–age–physiology

### VHA model and diagnostic performances

Based on the comparison of VHA results between the IPF/UIP and NSIP groups in cohort A, significant differences (adjusted *p* < 0.05) were observed in the kurtosis, skewness, and entropy values of the whole-lung, right upper lobe, and left upper lobe. Additionally, the entropy of the right lower lobe was also significantly different (Table 2). The kurtosis and skewness for UIP were significantly lower than those for NSIP, whereas the entropy for UIP was significantly higher than that for NSIP. These findings indicate that a classification model for UIP and NSIP patterns can be developed using VHA parameters obtained from individual lobes.

**Table 2:** Comparison between the Kurtosis, Skewness, and Entropy of IPF/UIP and NSIP Groups Classified by Multidisciplinary Discussion Diagnosis in cohort A

VHA Data	IPF/UIP Group	NSIP Group	*P* value
Whole-lung skewness	1.5 (1.3–1.9)	2.0 (1.6–2.3)	0.003
Whole-lung kurtosis	4.6 (4.0–6.4)	6.7 (5.0–8.4)	0.003
Whole-lung entropy	8.7 (8.4–8.9)	8.4 (8.1–8.6)	< 0.005
Right upper lobe skewness	1.8 (1.6–2.2)	2.6 (2.2–3.0)	< 0.005
Right upper lobe kurtosis	6.2 (5.2–7.9)	10.0 (7.9–12.9)	< 0.005
Right upper lobe entropy	8.5 (8.2–8.7)	8.0 (7.8–8.2)	< 0.005
Right middle lobe skewness	2.1 (1.5–2.4)	2.3 (1.7–2.8)	0.255
Right middle lobe kurtosis	7.1 (4.6–9.1)	8.2 (5.6–11.5)	0.321
Right middle lobe entropy	8.4 (8.1–8.7)	8.1 (7.8–8.5)	0.201
Right lower lobe skewness	1.2 (0.8–1.4)	1.4 (1.0–1.9)	0.177
Right lower lobe kurtosis	3.6 (2.7–4.1)	4.1 (3.1–6.3)	0.174
Right lower lobe entropy	9.0 (8.8–9.2)	8.7 (8.4–9.0)	0.015
Left upper lobe skewness	1.7 (1.5–2.4)	2.4 (1.9–2.7)	< 0.005
Left upper lobe kurtosis	5.8 (4.6–8.4)	9.0 (6.3–10.4)	< 0.005
Left upper lobe entropy	8.6 (8.2–8.8)	8.1 (7.9–8.4)	< 0.005
Left lower lobe skewness	1.3 (0.8–1.6)	1.4 (1.0–1.9)	0.762
Left lower lobe kurtosis	3.8 (2.8–5.0)	4.3 (3.1–6.2)	0.645
Left lower lobe entropy	8.9 (8.6–9.1)	8.7 (8.4–8.9)	0.132

Values are expressed as median (25th–75th percentile of the interquartile range). *P* values were calculated using the Mann–Whitney U test and subsequently adjusted for multiple comparisons using the Bonferroni method for the three families of metrics (kurtosis, skewness, and entropy). An adjusted *p* value < 0.05 was considered statistically significant. The adjusted *p* values are shown in the table. The NSIP group consisted of patients with idiopathic nonspecific interstitial pneumonia (iNSIP) and connective tissue disease-associated nonspecific interstitial pneumonia (CTD-NSIP)

VHA volume histogram analysis, IPF idiopathic pulmonary fibrosis, UIP usual interstitial pneumonia

**Fig. 2:**
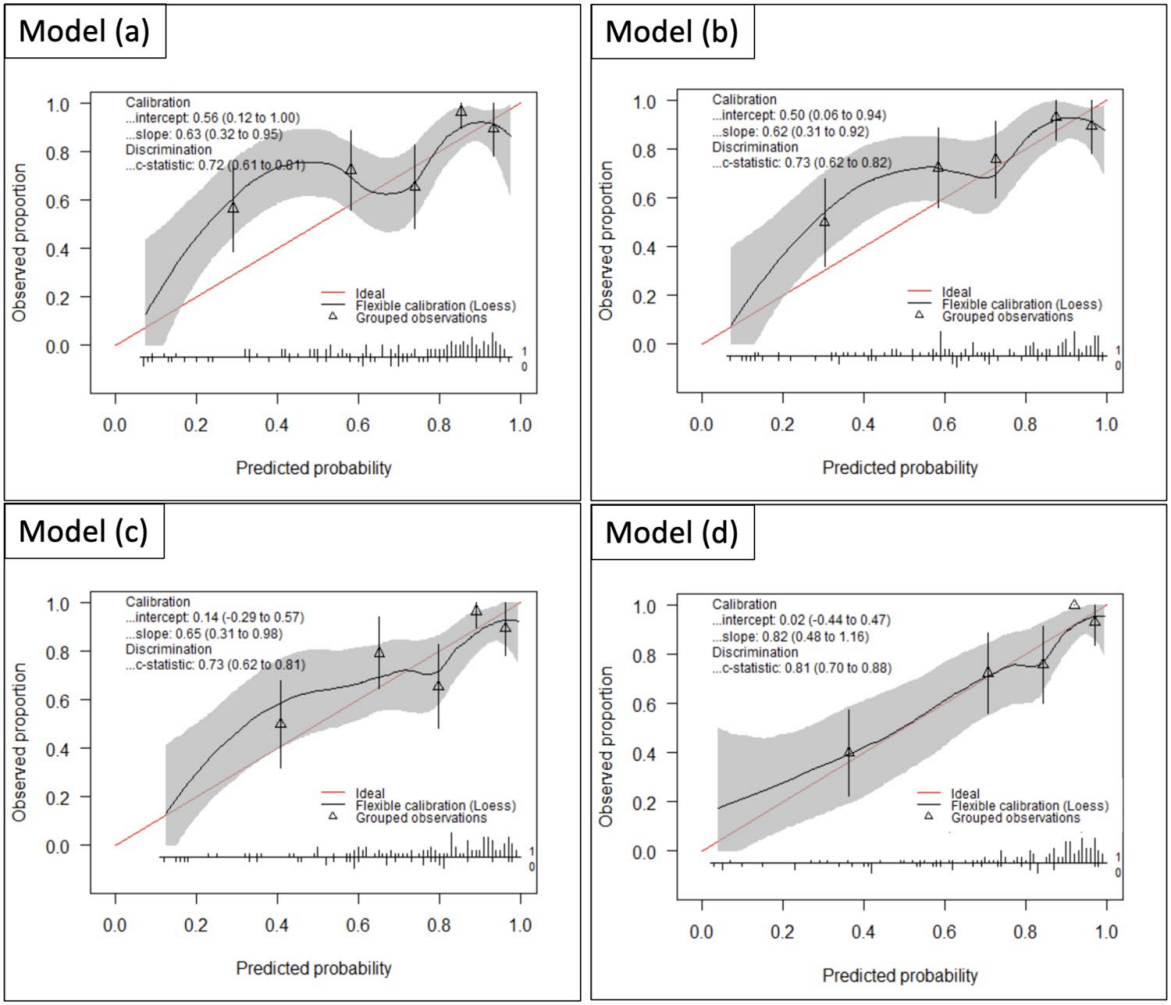
The AUC values and calibration plots are the results of the external validation for each model: models (**a**) to (**d**). Model (**d**) shows the best AUC values and the highest correlation with the calibration line (identity line = red line). Four models were developed based on different data inputs: **a** only kurtosis data, **b** only skewness data, **c** only entropy data, and **d** all three data types (kurtosis, skewness, and entropy). Vertical axis: observed proportion. Horizontal axis: predicted probability. The red line (ideal) represents the ideal calibration line in which the predicted probabilities accurately match the observed proportions. The black line (flexible calibration [Loess]) represents the smoothed calibration curve using local regression (Loess), showing the relationship between the predicted probabilities and observed proportions. The triangles (grouped observations) represent grouped observations, with error bars showing the variability within each group. AUC area under the receiver operating characteristic curve, CI confidence interval

**Fig. 3:**
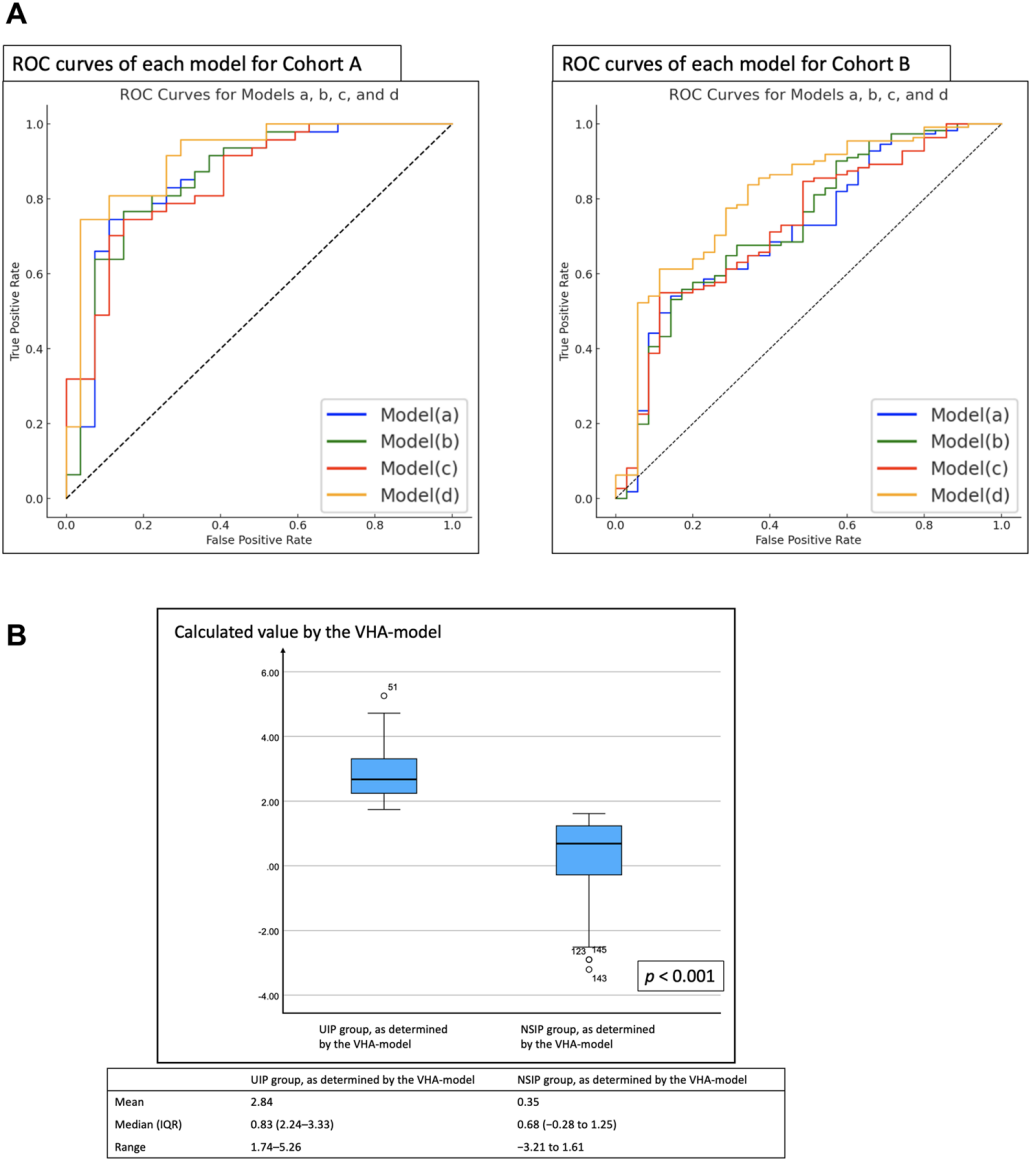
Figure 3A shows the ROC curves of each model for cohort A and cohort B. The result is considered a true positive when each model: models (**a**) to (**d**) correctly diagnoses IPF/UIP patterns and a true negative when it correctly diagnoses NSIP patterns. Model (**d**), which achieved the highest AUC, was designated as “the VHA model”. Four models were developed based on different data inputs: **a** only kurtosis data, **b** only skewness data, **c** only entropy data, and **d** all three data types (kurtosis, skewness, and entropy). Figure 3B shows the box-and-whisker diagram while adapting the cutoff value (1.68) calculated from the VHA model to cohort B. Groups identified as having UIP patterns and NSIP patterns by the VHA model were unambiguously classified by the calculated cutoff values. IQR represents the interquartile range (25th–75th percentile). ROC Receiver Operating Characteristic, VHA volume histogram analysis, IPF idiopathic pulmonary fibrosis, UIP usual interstitial pneumonia, NSIP nonspecific interstitial pneumonia

In the five-fold cross-validation with cohort A, the AUC was 0.86 (95% CI, 0.76–0.96) for model (a), 0.84 (0.74–0.94) for model (b), 0.85 (0.76–0.94) for model (c), and 0.91 (0.84–0.98) for model (d). These results identified model (d) as the top performer (Table 3). In the external validation with cohort B, the AUC was 0.72 (0.61–0.81) for model (a), 0.73 (0.62–0.82) for model (b), 0.73 (0.62–0.81) for model (c), and 0.81 (0.70–0.88) for model (d) (Table 3, Fig. 2). The corresponding ROC curves are shown in Fig. 3A. The calibration plots indicated that model (d) exhibited the best calibration line (identity line). The validation cohort was assessed using model (d) (Fig. 2). Based on these results, we designated model (d) as “the VHA model.” The detailed formula for the VHA model is given in Appendix S5.

**Table 3:** Results of the Internal Validation and External Validation for Each Model

	Internal validation: Cohort A			External validation: Cohort B	
Models	AUC	95% CI		AUC	95% CI
Model (a)	0.86	0.76–0.96		0.72	0.61–0.81
Model (b)	0.84	0.74–0.94		0.73	0.62–0.82
Model (c)	0.85	0.76–0.94		0.73	0.62–0.81
Model (d)	0.91	0.84–0.98		0.81	0.70–0.88

Four models were developed using histogram analysis data from each lung lobe: model (a) based on only kurtosis data, model (b) only skewness data, model (c) only entropy data, and model (d) all three data types (kurtosis, skewness, and entropy). Model (d) shows the highest AUC and is hereafter referred to as “the VHA model”. For AUC calculation, if a case diagnosed as IPF/UIP by MDD was classified as UIP by each model, it was considered a true positive. Similarly, if a case diagnosed as NSIP by MDD was classified as NSIP by each model, it was considered a true negative

AUC area under the receiver operating characteristic curve, CI confidence interval. VHA volume histogram analysis, IPF idiopathic pulmonary fibrosis, UIP usual interstitial pneumonia, MDD multidisciplinary discussion, NSIP nonspecific interstitial pneumonia

**Fig. 4:**
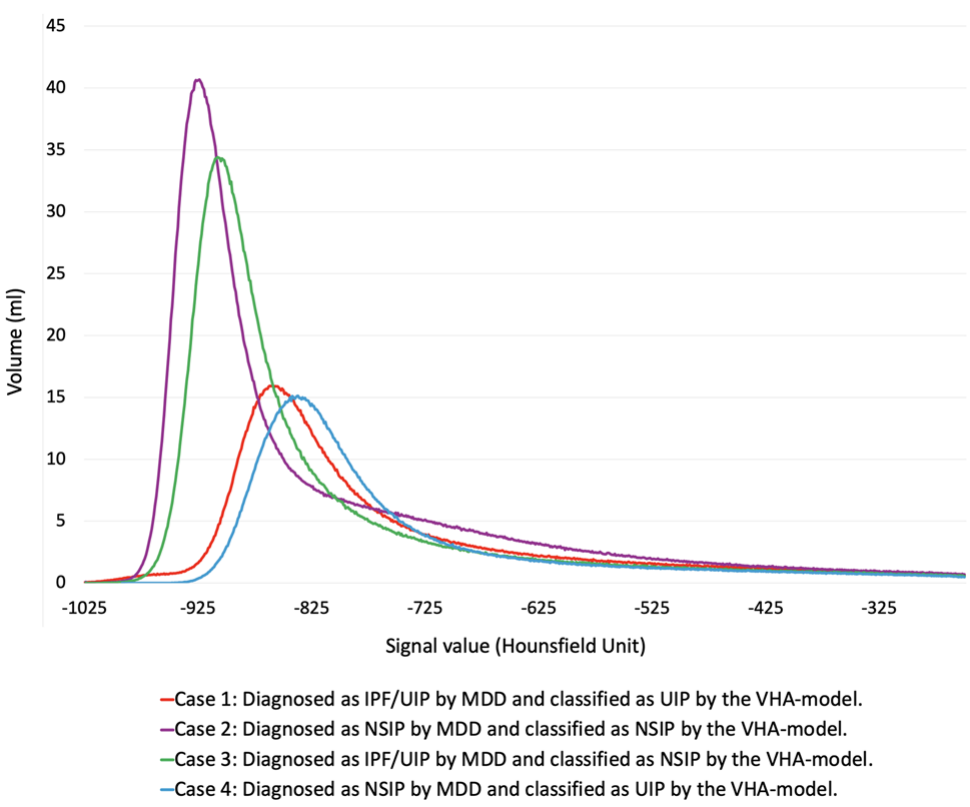
Whole-lung volume histogram plots for four representative cases. This figure shows the whole-lung histograms generated from the analysis of the CT images in these four representative cases. Cases 1 and 4 show histograms with lower skewness and kurtosis, whereas Cases 2 and 3 show higher skewness and kurtosis. These differences in the shape of the histogram curve reflect the degree of variation and complexity associated with voxel attenuation across the lung parenchyma. Case 1: Diagnosed as IPF/UIP by MDD and classified as UIP by the VHA model. Case 2: Diagnosed as NSIP by MDD and classified as NSIP by the VHA model. Case 3: Diagnosed as IPF/UIP by MDD and classified as NSIP by the VHA model. Case 4: Diagnosed as NSIP by MDD and classified as UIP by the VHA model. The VHA model refers to a model formulated based on the kurtosis, skewness, and entropy obtained from histogram analysis of each lung lobe. IPF idiopathic pulmonary fibrosis, UIP usual interstitial pneumonia, MDD multidisciplinary discussion, NSIP nonspecific interstitial pneumonia, VHA volume histogram analysis, CT computed tomography

The cutoff value for differentiating UIP patterns from NSIP patterns using the VHA model was 1.68. The calculated value was derived from the predicted probability, which was logit-transformed. Figure 3B presents box-and-whisker plots in which this cutoff value was used to classify cohort B patients based on UIP and NSIP patterns. UIP and NSIP groups identified by the VHA model were unambiguously classified using the calculated cutoff values. Table 4 presents the diagnostic performance of the VHA model in differentiating UIP patterns from NSIP patterns. In cohort A, the VHA model achieved a sensitivity of 0.75 (95% CI: 0.68–0.76), a specificity of 0.96 (95% CI: 0.85–0.99), an accuracy of 0.82 (95% CI: 0.74–0.85), a PPV of 0.97 (95% CI: 0.88–1.00), a NPV of 0.68 (95% CI: 0.60–0.71), and an AUC of 0.91 (95% CI: 0.84–0.98). In cohort B, the VHA model achieved a sensitivity of 0.61 (95% CI: 0.57–0.63), a specificity of 0.89 (95% CI: 0.76–0.95), an accuracy of 0.68 (95% CI: 0.62–0.71), a PPV of 0.94 (95% CI: 0.88–0.98), an NPV of 0.42 (95% CI: 0.36–0.45), and an AUC of 0.81 (95% CI: 0.70–0.88). Representative CT images of typical and borderline UIP/NSIP are shown in Figure S2 (corresponding VHA results in Table S1), and whole-lung histograms for the same cases are shown in Fig. 4.

**Table 4:** Diagnostic Performance of the VHA-Model in Each cohort

	Cohort A			Cohort B	
		95% CI			95% CI
Sensitivity	0.75	0.68–0.76		0.61	0.57–0.63
Specificity	0.96	0.85–0.99		0.89	0.76–0.95
Accuracy	0.82	0.74–0.85		0.68	0.62–0.71
Positive predictive value	0.97	0.88–1.00		0.94	0.88–0.98
Negative predictive value	0.68	0.60–0.71		0.42	0.36–0.45
AUC	0.91	0.84–0.98		0.81	0.70–0.88

The result is considered true positive when the VHA model correctly diagnoses IPF/UIP patterns and true negative when it correctly diagnoses NSIP patterns. The VHA model refers to a model formulated based on the kurtosis, skewness, and entropy obtained from histogram analysis of each lung lobe. Cohort A refers to the cohort used for internal validation. Cohort B refers to the cohort used for external validation

VHA volume histogram analysis, CI confidence interval, IPF idiopathic pulmonary fibrosis, UIP usual interstitial pneumonia, NSIP nonspecific interstitial pneumonia, AUC area under the receiver operating characteristic curve

### Follow-up duration and mortality in cohort B

Cohort B consisted of 111 patients with IPF/UIP, 30 with iNSIP, and 5 with CTD-NSIP. During the follow-up period, deaths occurred in 41 of 111 patients with IPF/UIP (37%), 3 of 30 with iNSIP (10%), and 0 of 5 with CTD-NSIP. The median follow-up duration was 1257 (IQR, 672–1585) days for the IPF/UIP group, 1224 (IQR, 464–1685) days for the iNSIP group, and 1014 (IQR, 390–1222) days for the CTD-NSIP group. Kruskal–Wallis H test showed no statistically significant difference in the follow-up durations among these three groups (*p* = 0.367).

### Relationship between VHA model and prognosis

**Fig. 5:**
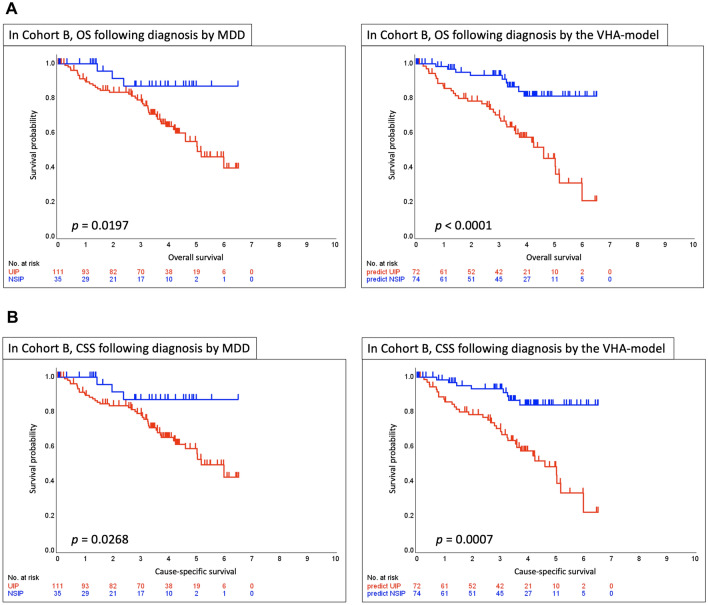
Kaplan–Meier survival curves for OS and CSS based on diagnosis by MDD and the VHA model in cohort B. **A** Kaplan–Meier survival curves based on OS. **B** Kaplan–Meier survival curves based on CSS. The results indicate that the group diagnosed with IPF/UIP by the VHA model has worse prognosis for OS and CSS than the group diagnosed with IPF/UIP by MDD. The VHA model refers to a model formulated based on the kurtosis, skewness, and entropy obtained from histogram analysis of each lung lobe. OS overall survival, CSS cause-specific survival, MDD multidisciplinary discussion, IPF idiopathic pulmonary fibrosis, UIP usual interstitial pneumonia, NSIP nonspecific interstitial pneumonia

Using Kaplan–Meier survival curves derived from MDD diagnosis and model determination, patients diagnosed with IPF/UIP via MDD had significantly worse OS than those diagnosed with NSIP (*p* = 0.0197). Similarly, patients identified as having UIP by the VHA model had significantly worse OS than those diagnosed with NSIP (*p* < 0.0001). Regarding CSS, the diagnosis of IPF/UIP via MDD was associated with significantly worse survival than the diagnosis of NSIP (*p* = 0.0268). Similarly, the VHA model–based diagnosis indicated that patients with UIP had significantly worse CSS than those with NSIP (*p* = 0.0007) (Fig. 5). In MDD-diagnosed cases with UIP, patients classified as UIP by the VHA model showed significantly worse OS (*p* = 0.0036) and CSS (*p* = 0.0025) than those classified as NSIP. Similarly, in MDD-diagnosed cases with NSIP, VHA-based UIP status indicated significantly worse OS (*p* = 0.0116) and CSS (*p* = 0.0116). The Kaplan–Meier curves for these comparisons are shown in Figure S3.

### Cox regression analysis adjusted for clinical variables

After adjustment for age, sex, pulmonary function tests (%FVC and %DLCO), smoking history, and institutional diagnosis, the calculated values in the VHA model were significantly correlated with both OS (hazard ratio [HR] = 1.60; 95% CI: 1.17–2.18; *p* = 0.003) and CSS (HR = 1.70; 95% CI: 1.24–2.35; *p* = 0.001). Similarly, after adjustment for GAP score, smoking history, and institutional diagnosis, the correlations with OS (HR = 1.56; 95% CI: 1.16–2.09; *p* = 0.003) and CSS (HR = 1.64; 95% CI: 1.21–2.23; *p* = 0.001) remained significant.

## Discussion

In this study, we developed a formula-based model using volume histogram analysis (VHA) of whole-lung computed tomography (CT) data from each lung lobe to differentiate usual interstitial pneumonia (UIP) from nonspecific interstitial pneumonia (NSIP). In internal validation, the VHA model achieved an area under the receiver operating characteristic curve (AUC) of 0.91, with high performance metrics including a sensitivity of 0.75, specificity of 0.96, and accuracy of 0.82, and demonstrated an excellent positive predictive value (PPV) of 0.97. External validation showed an AUC of 0.81, with a sensitivity of 0.61, a specificity of 0.89, an accuracy of 0.68, and a PPV of 0.94. Additionally, patients classified as UIP by the VHA model had significantly worse overall survival (OS) (*p* < 0.0001) and cause-specific survival (CSS) (*p* = 0.0007) than those classified as NSIP.

Several deep learning models trained on chest CT images have emerged in the field of image analysis of interstitial pneumonia [12–14]. A recent report by Koo et al. indicated that machine learning models using quantitative CT features classified UIP and non-UIP patterns with an AUC of 0.88 [17], whereas the lung intelligence kit used by Chen et al. demonstrated an ability to differentiate UIP patterns from NSIP patterns with an AUC of 0.838 [18]. A common issue with deep learning models is the lack of clarity in the algorithms employed. Our formula-based VHA model demonstrated better performance in distinguishing UIP compared to previously reported results in internal validation (AUC, 0.91), and based on external validation at another hospital, where differentiation was more difficult, the performance of this model in differentiating UIP was also relatively high (AUC, 0.81).

In UIP, fibrosis is heterogeneous, and various attenuation values exist randomly, leading to relatively lower kurtosis and skewness than those in NSIP, which has uniform fibrosis and less variability in attenuation values. As entropy reflects the complexity of fibrosis, the entropy of UIP is considered higher than that of NSIP. This difference in VHA data between the UIP and NSIP patterns was shown in a comparative analysis of whole-lung and lobe units performed preliminarily in cohort A. However, only a few studies have compared UIP and NSIP patterns using histogram analysis. Do et al. [21] evaluated CT images 1 cm above the diaphragm in a comparative analysis of UIP without honeycombing and NSIP patterns and revealed that UIP had higher kurtosis and skewness than NSIP. Tanizawa et al. [22] compared IPF with a group of non-IPF interstitial pneumonias, including NSIP, and found no differences in kurtosis and skewness. Sumikawa et al. [19] reported that when using VHA with regions of interest set to the entire lung, right lower lobe, and small cubic volume of interest (VOI), the entropy in the cubic VOI only was significantly higher in UIP than in NSIP. When comparing VHA results between UIP and NSIP, variations have been observed in the results of kurtosis, skewness, and entropy between previous studies and our study. These discrepancies may be attributed to differences in the progression of interstitial pneumonia and definitions of region of interest or VOI. Notably, Sumikawa et al. used VOI for VHA and reported higher entropy for UIP than for NSIP, consistent with our results. This consistency highlights the reliability of VOI-based approaches for distinguishing entropy differences between these interstitial pneumonia patterns, despite methodological differences. If the differentiation between UIP and NSIP based on the kurtosis, skewness, and entropy of each lung lobe was based on these considerations, it could be argued that this model offers a certain perspective on explainability in artificial intelligence diagnosis. Furthermore, as kurtosis, skewness, and entropy are all useful factors in differentiating UIP from NSIP patterns, it was considered that, of the four models developed in this study, the model using all three factors (i.e., kurtosis, skewness and entropy) exhibited the best discriminatory ability. Our results also showed that upper lobe kurtosis, skewness, and entropy differed significantly between UIP and NSIP, suggesting more pronounced structural changes in upper lobes of the lungs in UIP. This is consistent with the findings of Hunninghake et al. [29] and Gruden et al. [30] who suggested that upper lobe involvement is characteristic of UIP even though it may be less severe than that in the lower zones and believe the presence of some upper lobe involvement is essential to the diagnosis. By analyzing each lobe separately, our VHA model may better detect such upper lobe fibrotic changes, improving its diagnostic ability.

When cohort B was classified using this model, approximately 39% (43/111) of patients diagnosed with IPF/UIP via MDD had an NSIP, not UIP, pattern. This finding is relatively close to the values reported in previous studies, demonstrating that among patients with a pathological diagnosis of UIP and a clinical diagnosis of IPF, 29% (29/98) [31] to 32% (24/75) [32] showed radiological NSIP or unclassifiable pattern but not UIP pattern.

Although the diagnostic accuracy of our VHA model in the external validation was 0.68, it achieved an excellent PPV of 0.94. This finding has significant implications for the model’s clinical utility. In a real-world setting, the prevalence of diseases within the spectrum of IIPs has been reported to be 47–64% for IPF and 14–36% for NSIP [33]. In a prospective survey of IIPs in a web registry in Japan, among the 436 patients initially registered with IIPs, 321 (74%) were IPF, and 82 (19%) were NSIP [34]. The composition of cohort B in our study, with 111 cases of IPF/UIP (76%) and 35 cases of NSIP (24%), reflects this clinical reality in which IPF/UIP constitutes the majority of cases. In scenarios with such imbalanced prevalence, relying solely on accuracy as a performance metric has limitations. Instead, a high PPV is important for diagnosing IPF/UIP, a condition with a poorer prognosis that requires specific therapeutic interventions.

In cohort B, patients classified as having a UIP pattern (i.e., IPF) by the VHA model had a worse prognosis than those diagnosed with IPF via MDD. These findings suggest that determining UIP patterns using the VHA model can extract cases with worse prognosis. Specifically, the model classifies UIP when entropy (complexity of fibrosis) is high and kurtosis and skewness (uniformity of fibrosis) are low, characteristics indicative of heterogeneous and advanced fibrosis. In Cox analysis adjusted for clinical variables, the values calculated using the VHA model correlated significantly with prognosis. Furthermore, even among IPF/UIP or NSIP via MDD, those reclassified as UIP by the VHA model had significantly worse OS and CSS than those classified as NSIP. This result indicated that if the value calculated by this model exceeded the cutoff value calculated in this study, the case was classified as interstitial pneumonia with a worse prognosis and required more stringent therapeutic intervention.

Based on the abovementioned results, the model developed in this study may help diagnose CT UIP patterns, particularly for identifying UIP patterns associated with poor prognosis.

This study had several limitations. This was a retrospective study, and matching relevant patient background data within each cohort and between cohorts was difficult. Matching the degree of fibrosis between IPF/UIP and NSIP cases was impossible. Discrepancies in patient background and degree of fibrosis may have affected the prognostic analysis. The NSIP group comprised iNSIP and CTD-NSIP, but the NSIP group in validation cohort B comprised predominantly iNSIP. No deaths occurred in the CTD-NSIP subgroup during follow-up, and follow-up duration was comparable within the NSIP subgroups. Therefore, the inclusion of a small CTD-NSIP subgroup is unlikely to have materially affected our prognostic analysis. Differences in CT conditions between cohorts A and B may have affected the results of VHA and accuracy of the VHA model presented in this study. The automatic lung extraction function used in this study may not have recognized consolidation as a lung lesion or incorrectly recognized pulmonary arteries and veins as lung tissue. Therefore, our method may not be feasible in patients with extensive consolidation or inadequate inspiratory effort.

In conclusion, we developed a formula-based model to discriminate radiological usual interstitial pneumonia patterns with high discriminatory power using volume histogram analysis (i.e., kurtosis, skewness, and entropy) of whole-lung CT volumetric data obtained from each lung lobe. Unlike previous methods, the current approach targets fibrosis in each lobe and may help identify patients with interstitial pneumonia who have poor prognosis. Future studies will involve validating the model in large-scale prospective cohorts and exploring its utility for other interstitial lung disease patterns and for secondary interstitial pneumonias as well as idiopathic ones. Furthermore, in future, how this model works in longitudinal CT follow-up studies of patients with fibrosis should be evaluated because it is hard to accurately evaluate disease progression solely based on visual assessment.

## Supplementary Information

Below is the link to the electronic supplementary material.

Supplementary file1 (DOCX 6749 KB)
