# Supplementary material for: Volume histogram analysis of whole-lung CT: differentiating usual from nonspecific interstitial pneumonias and predicting prognosis
Source: Jpn J Radiol. 2025 Oct 10;44(2):291–302. doi: 10.1007/s11604-025-01880-9 (PMC12860808; doi:10.1007/s11604-025-01880-9)
Supplement: Supplementary file 1 — Supplementary file1 (DOCX 6749 KB) [file 11604_2025_1880_MOESM1_ESM.docx]

Supplemental Material

**Appendix S1: The Patient Selection Process**

This study included consecutive adult patients with usual interstitial pneumonia (UIP) pattern of idiopathic pulmonary fibrosis (IPF) and those with nonspecific interstitial pneumonia (NSIP) pattern of idiopathic NSIP (iNSIP) and connective tissue disease-associated NSIP (CTD-NSIP). All patients underwent multidisciplinary discussion (MDD) between April 2014 and March 2021, at our university hospital (Cohort A) and at a collaborating hospital (Cohort B). In each cohort, if there were computed tomography (CT) findings (e.g., bacterial pneumonia with extensive consolidation or lung collapse and extensive multiple large cysts) that were clearly inconsistent with IPF/UIP or NSIP, these cases were excluded based on a central review of the imaging.

In Cohort A, 63 patients (50 men and 13 women) were initially diagnosed with IPF/UIP. Of these, 16 patients were excluded for the following reasons: findings from a central review of imaging showing findings clearly inconsistent with IPF/UIP (five men and one woman); CT scans not performed at our hospital (seven men and two women); errors in reading the CT data (one man). After these exclusions, 47 patients (37 men and 10 women) remained with IPF/UIP. Similarly, 31 patients (11 men and 20 women) were initially identified in the NSIP group. Of these, four patients were excluded for the following reasons: findings from a central review of imaging showing findings clearly inconsistent with NSIP (one man); CT scans not performed at our hospital (one man and two women). After these exclusions, 27 patients (nine men and 18 women) remained with NSIP patterns.

In Cohort B, 117 patients (92 men and 25 women) were initially diagnosed with IPF/UIP. Of these, six patients were excluded for the following reasons: findings from a central review of imaging showing findings clearly inconsistent with IPF/UIP (five men); errors in reading the CT data (one man). After these exclusions, 111 patients (86 men and 25 women) remained with IPF/UIP. Similarly, 43 patients (21 men and 22 women) were initially identified in the NSIP group. Of these, eight patients were excluded for the following reasons: findings from a central review of imaging showing findings clearly inconsistent with NSIP (two men and five women); errors in reading the CT data (one man). After these exclusions, 35 patients (18 men and 17 women) remained with NSIP patterns.

**Appendix S2: Details of the Disease Composition of the IPF/UIP and NSIP Pattern Groups in Each Cohort**

**IPF/UIP Group**

In Cohort A, of the 47 patients with idiopathic pulmonary fibrosis (IPF)/usual interstitial pneumonia (UIP) 45 had a definite UIP with honeycombing, and two had a probable UIP. These two patients with a probable UIP underwent surgical lung biopsy (SLB), which pathologically confirmed IPF/UIP. In Cohort B, of the 111 patients with IPF/UIP, 81 were diagnosed based on clinical information and imaging findings, while the remaining 30 were diagnosed with IPF/UIP by SLB. A central review of all 111 cases was conducted. Among the cases diagnosed by clinical and imaging findings, 72 were classified as definite UIP and 9 as probable UIP. In all cases that underwent SLB, there were suggestive findings of airway disease/airway-centered fibrosis on CT; however, the main framework of fibrosis showed a UIP pattern. Among these, 21 had findings corresponding to a definite UIP pattern with honeycombing, and 9 had findings corresponding to a probable UIP pattern.

**NSIP Pattern Group**

The disease composition of the groups with nonspecific interstitial pneumonia (NSIP) patterns in each cohort was as follows. In Cohort A, there were six cases of idiopathic NSIP (iNSIP) and 21 cases of connective tissue disease-associated NSIP (CTD-NSIP) (15 cases of dermatomyositis, three cases of clinically amyopathic dermatomyositis, two cases of polymyositis, and one case of Sjögren’s syndrome). In Cohort B, there were 30 cases of iNSIP and five cases of CTD-NSIP (four cases of systemic sclerosis and one case of Sjögren’s syndrome).

**Appendix S3: Details of Whole-Lung CT Scans**

For both cohorts, whole-lung volumetric thin-section computed tomography (CT) scans were performed, encompassing areas from the apex to the base of the lung in the supine position with full inspiration using various CT scanners. The tube voltage was set to 120 kV, and the tube current was set to “Auto.” In Cohort A, CT was performed using detectors with 64–256 rows and a slice thickness of 1.25 mm. The CT systems used in Cohort A included iCT® (Philips Healthcare, Amsterdam, the Netherlands), 750HD®, Revolution CT® and Revolution Apex® (all from GE Medical Systems, Chicago, IL, USA). In Cohort B, detectors with 16–80 rows were used, with a slice thickness of 1.00–1.25 mm. The CT systems used in Cohort B included Aquilion Prime® (Toshiba Medical Systems, Tokyo, Japan) and Light Speed16® (GE Medical Systems, Chicago, IL, USA). No reconstructions using high spatial frequency algorithms were performed, and intravenous contrast administration was not performed to avoid any effects on VHA. All CT scans were performed within one month of the initial diagnosis of interstitial pneumonia.

**Appendix S4: The Main Parameters Measured in Respiratory Function Tests**

Pulmonary function tests were performed using standard methods. The main parameters measured in respiratory function tests were as follows: forced vital capacity (FVC), percent predicted FVC, forced expiratory volume in one second (FEV1), FEV1 percent (FEV1/FVC ratio), diffusing capacity of the lungs for carbon monoxide (DLCO), and percent predicted DLCO. Data were retrospectively obtained from tests performed closest to the date of the CT used for histogram analyses.

**Appendix S5. The Equation of the Final VHA-Model for Differentiating UIP from NSIP**

y = − 71.23 − 0.39 × A + 0.57 × B + 1.54 × C − 0.25 × D + 1.24 × E + 4.52 × F − 0.45 × G − 0.18 × H + 2.81 × I + 2.99 × J

This equation was derived by Least Absolute Shrinkage and Selection Operator (LASSO) logistic regression using kurtosis, skewness, and entropy values from each lung lobe. Coefficients (−71.23 to +2.99) were estimated using training data in Cohort A. Parameters A–J are defined below.

| A | kurtosis of the right upper lobe |
| --- | --- |
| B | entropy of the right upper lobe |
| C | skewness of the right middle lobe |
| D | entropy of the right middle lobe |
| E | skewness of the right lower lobe |
| F | entropy of the right lower lobe |
| G | skewness of the left upper lobe |
| H | kurtosis of the left upper lobe |
| I | skewness of the left lower lobe |
| J | entropy of the left lower lobe |

Table S1. VHA Metrics and Model Output for the Four Representative Cases

| VHA Data | Case 1:  UIP/UIP | Case 2:  NSIP/NSIP | Case 3:  UIP/NSIP | Case 4:  NSIP/UIP |
| --- | --- | --- | --- | --- |
| Whole-lung skewness | 1.34 | 1.58 | 1.94 | 1.58 |
| Whole-lung kurtosis | 4.08 | 4.88 | 6.34 | 4.84 |
| Whole-lung entropy | 8.84 | 8.57 | 8.40 | 8.62 |
| Right upper lobe skewness | 1.92 | 2.19 | 2.45 | 1.44 |
| Right upper lobe kurtosis | 6.37 | 7.95 | 9.25 | 4.36 |
| Right upper lobe entropy | 8.41 | 8.23 | 8.03 | 8.68 |
| Right middle lobe skewness | 2.12 | 1.88 | 2.21 | 1.81 |
| Right middle lobe kurtosis | 7.44 | 6.06 | 7.90 | 5.81 |
| Right middle lobe entropy | 8.29 | 8.43 | 8.26 | 8.46 |
| Right lower lobe skewness | 1.23 | 0.57 | 1.61 | 1.40 |
| Right lower lobe kurtosis | 3.70 | 2.60 | 4.80 | 4.20 |
| Right lower lobe entropy | 8.91 | 9.27 | 8.60 | 8.76 |
| Left upper lobe skewness | 0.79 | 2.56 | 2.02 | 1.45 |
| Left upper lobe kurtosis | 2.81 | 9.78 | 6.80 | 4.26 |
| Left upper lobe entropy | 9.24 | 7.97 | 8.39 | 8.68 |
| Left lower lobe skewness | 0.90 | 0.69 | 1.40 | 1.89 |
| Left lower lobe kurtosis | 2.97 | 2.73 | 4.07 | 6.14 |
| Left lower lobe entropy | 9.13 | 9.18 | 8.75 | 8.39 |
| Model output | 3.04 | 0.23 | -0.09 | 3.00 |

Note. This table shows the quantitative VHA results for the whole lung and each lobe - namely skewness, kurtosis and entropy - for the four representative cases shown in Figure S2. The last row shows the result of applying these VHA results to the VHA-model’s formula, expressed to two decimal places with subsequent digits rounded. The VHA-model refers to a model formulated based on the kurtosis, skewness, and entropy obtained from histogram analysis of each lung lobe.

Case 1: UIP/UIP = Diagnosed as IPF/UIP by MDD and classified as UIP by the VHA-model.

Case 2: NSIP/NSIP = Diagnosed as NSIP by MDD and classified as NSIP by the VHA-model.

Case 3: UIP/NSIP = Diagnosed as IPF/UIP by MDD and classified as NSIP by the VHA-model.

Case 4: NSIP/UIP = Diagnosed as NSIP by MDD and classified as UIP by the VHA-model.

VHA volume histogram analysis, IPF idiopathic pulmonary fibrosis, UIP usual interstitial pneumonia, MDD multidisciplinary discussion, NSIP nonspecific interstitial pneumonia.


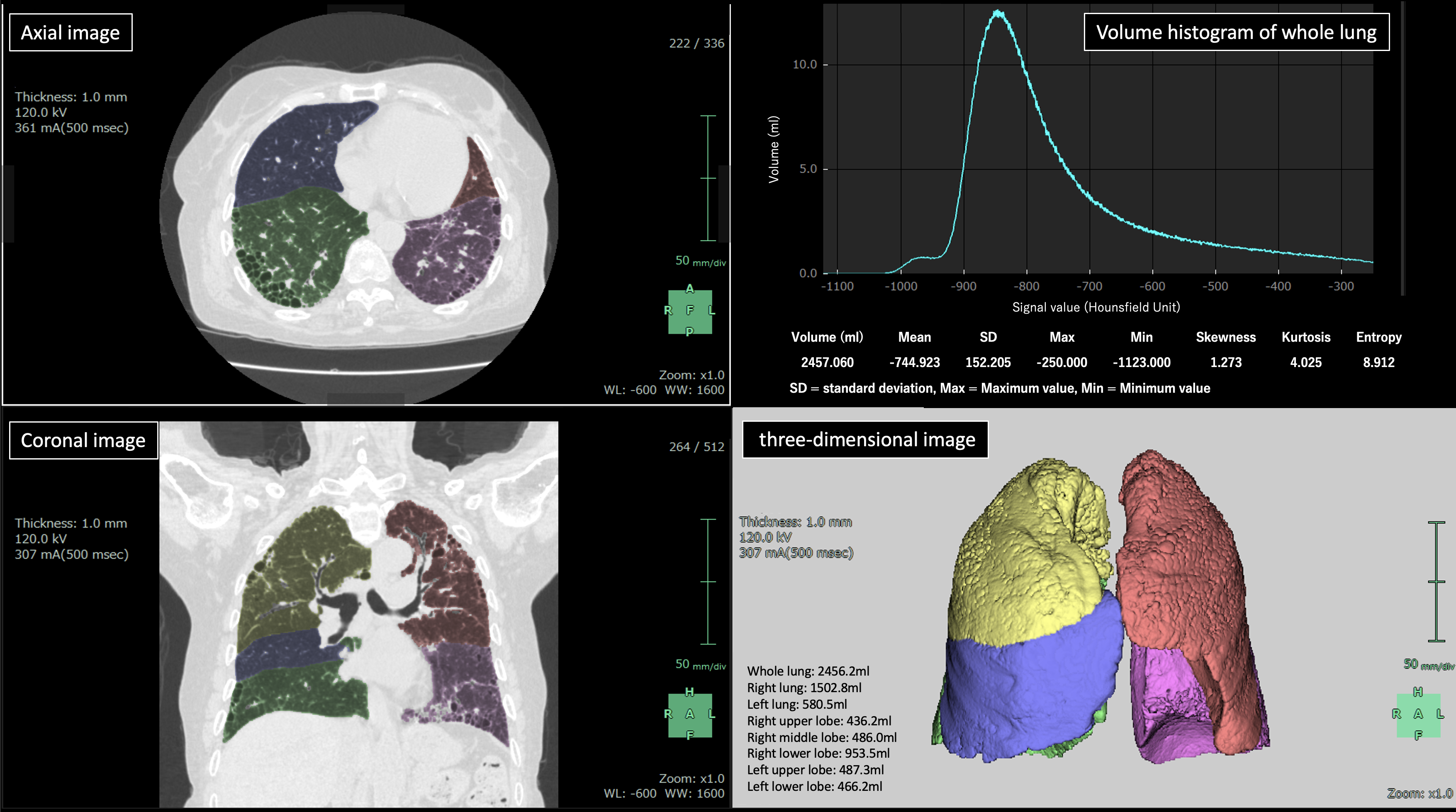


Figure S1. Image examples of the lung lobe segmentation and the VHA process using an actual case from the study.

These images illustrate how the software automatically segments each lung lobe and performs VHA in a patient with IPF/UIP diagnosed by MDD. The top left (axial) and bottom left (coronal) panels show color-coded lobar segmentation results. The upper right panel shows the volume histogram of the whole lung, including quantitative parameters such as volume, mean attenuation, standard deviation, skewness, kurtosis and entropy. The lower right panel shows a three-dimensional reconstruction of the automatically segmented lobes. Some changes have been made to the original screen, such as the translation of Japanese text into English.

VHA volume histogram analysis, IPF idiopathic pulmonary fibrosis, UIP usual interstitial pneumonia, MDD multidisciplinary discussion.


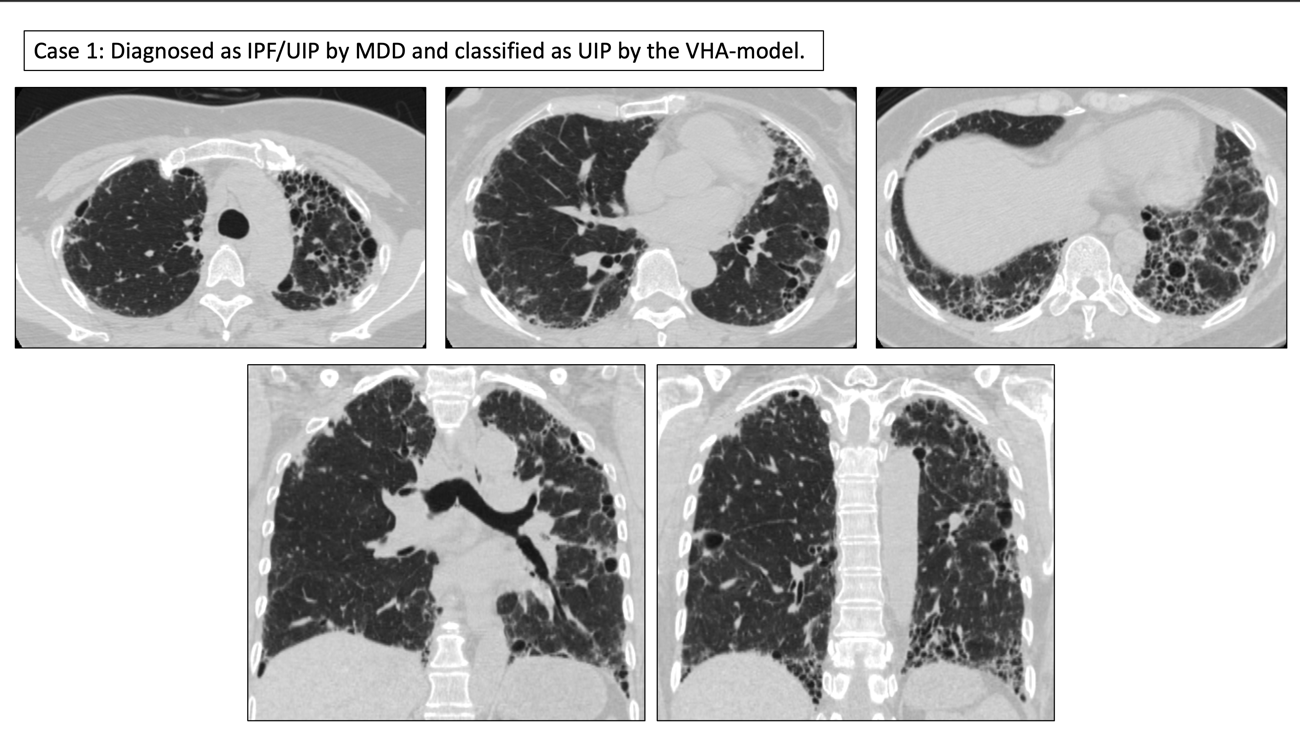


Figure S2A


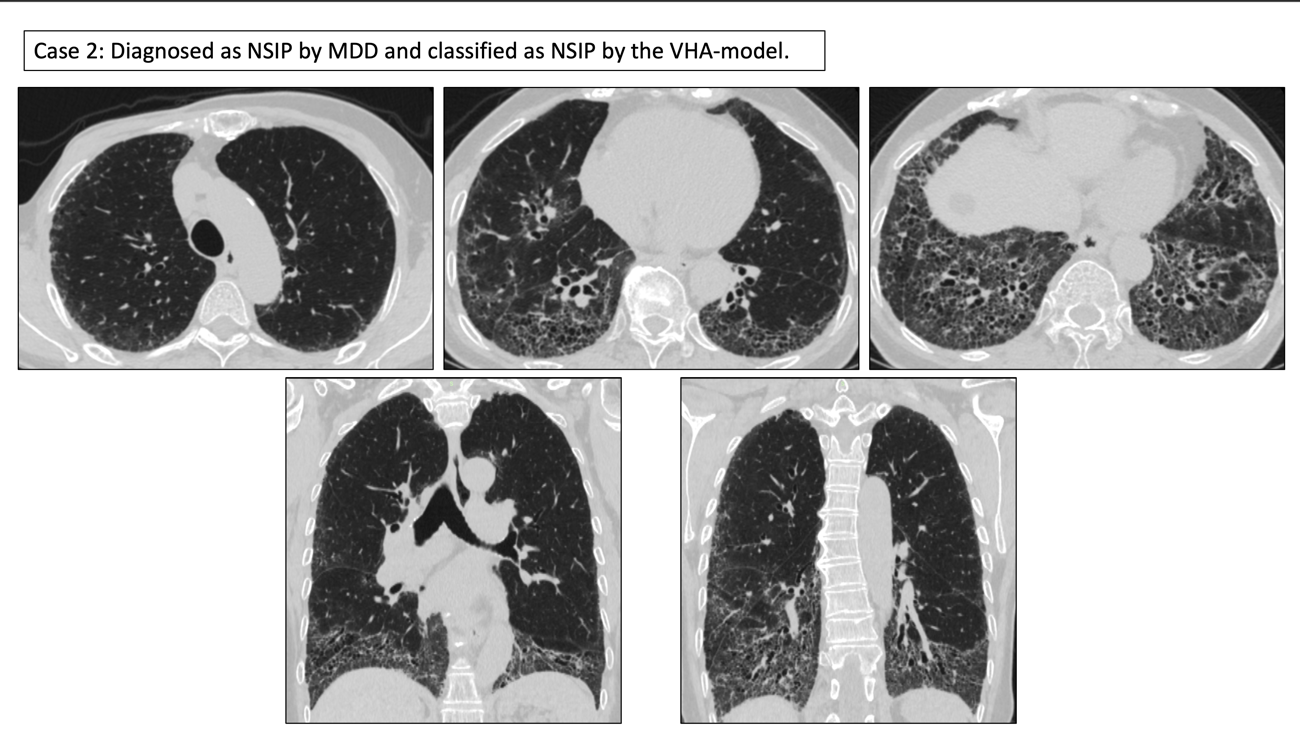


Figure S2B


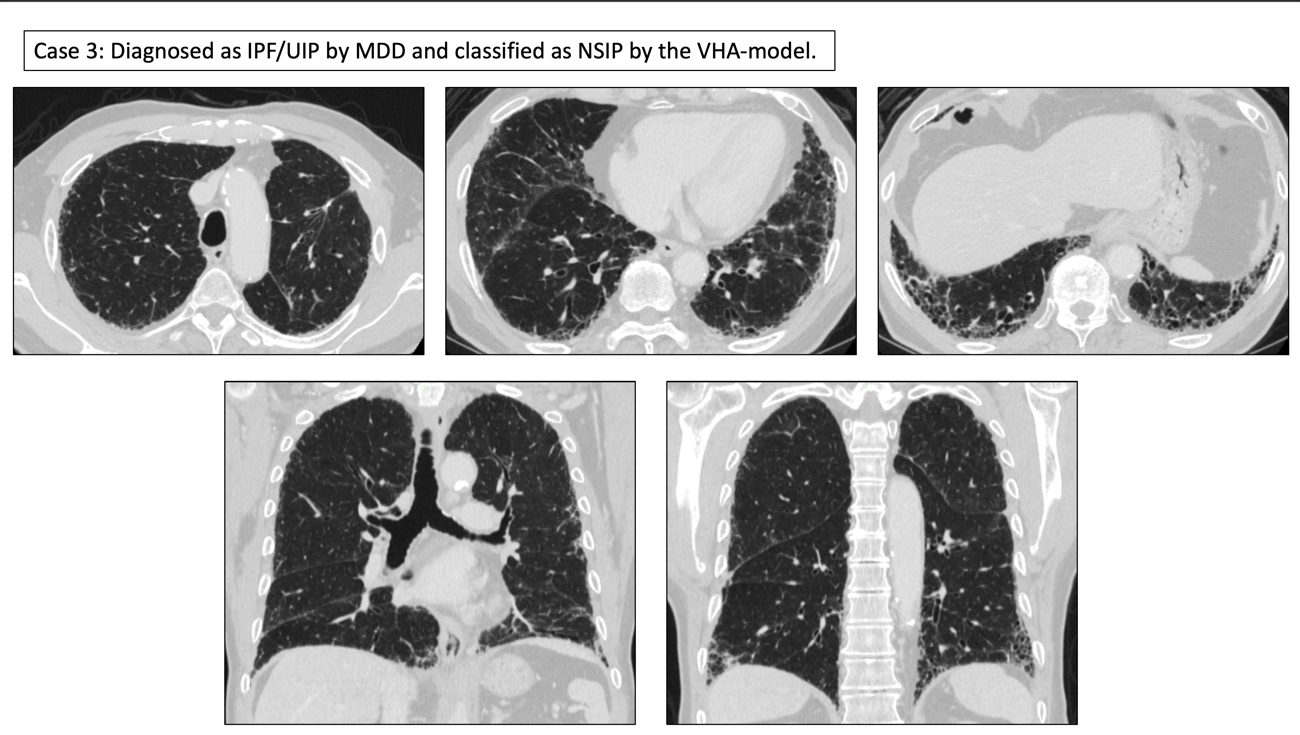


Figure S2C


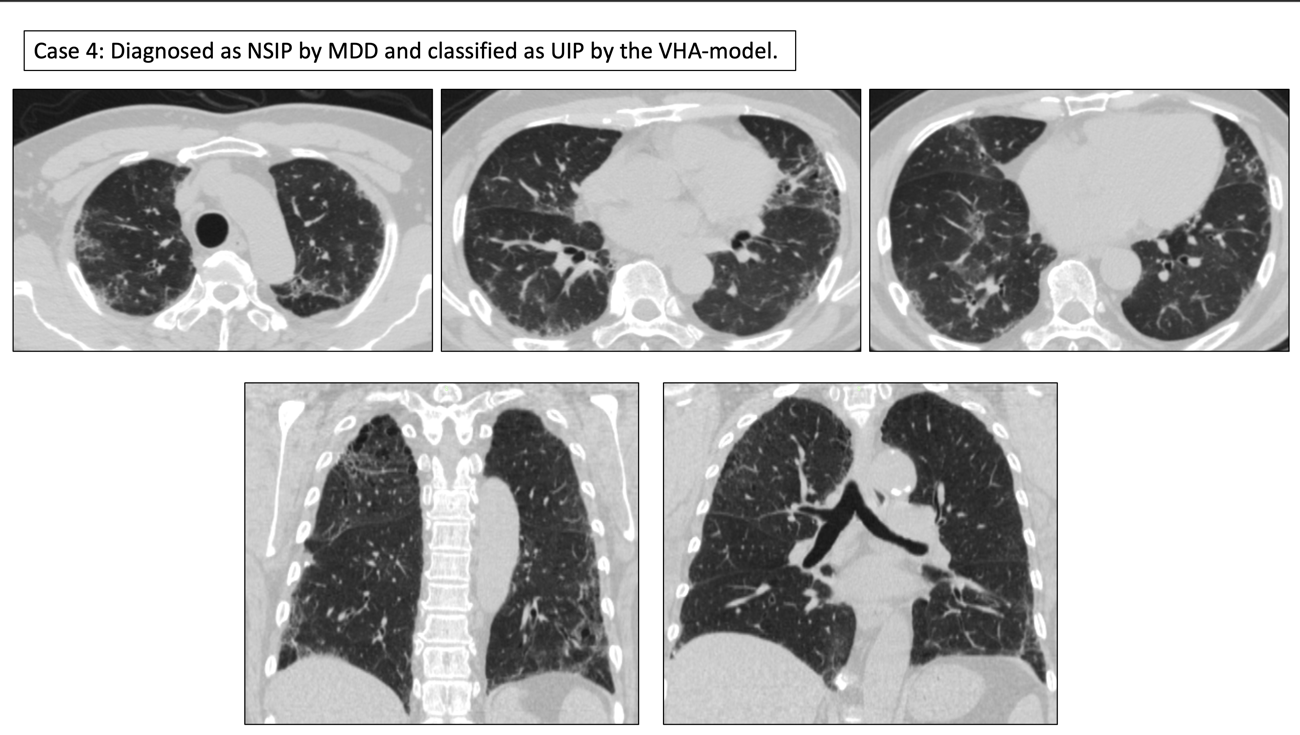


Figure S2D

Figure S2. Representative CT scans corresponding to the VHA‐Model classifications for four example cases in this study.

These images show axial (top row) and coronal (bottom row) CT slices from four patients in this study, highlighting typical and borderline presentations according to MDD diagnosis and the VHA-model output.

Case 1 (Figure S2A): Diagnosed as IPF/UIP by MDD and classified as UIP by the VHA-model.

Case 2 (Figure S2B): Diagnosed as NSIP by MDD and classified as NSIP by the VHA-model.

Case 3 (Figure S2C): Diagnosed as IPF/UIP by MDD and classified as NSIP by the VHA-model.

Case 4 (Figure S2D): Diagnosed as NSIP by MDD and classified as UIP by the VHA-model.

For each case, the corresponding volume histogram metrics (e.g., skewness, kurtosis, and entropy) of each lobe and the whole lung along with the final calculated outputs from the VHA-model formula are shown in Table S1. The VHA-model refers to a model formulated based on the kurtosis, skewness, and entropy obtained from histogram analysis of each lung lobe.

CT computed tomography, VHA volume histogram analysis, MDD multidisciplinary discussion, IPF idiopathic pulmonary fibrosis, UIP usual interstitial pneumonia, NSIP nonspecific interstitial pneumonia.


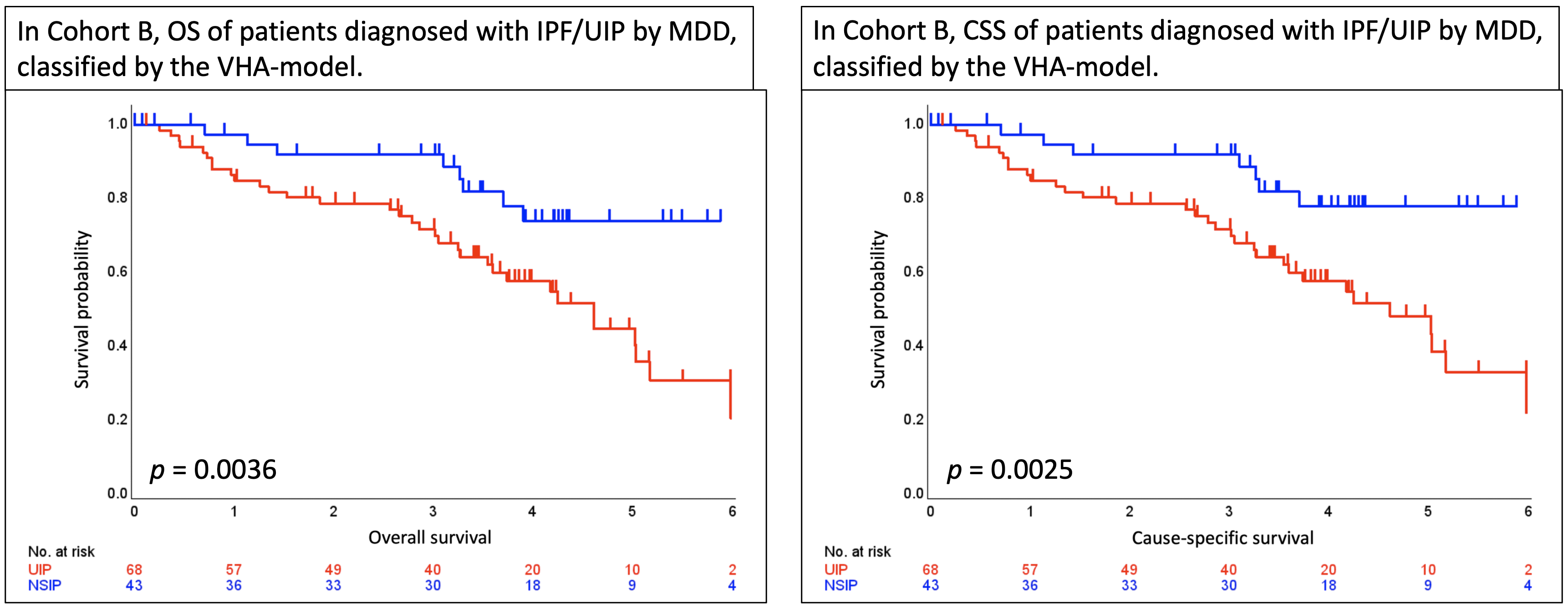


Figure S3A


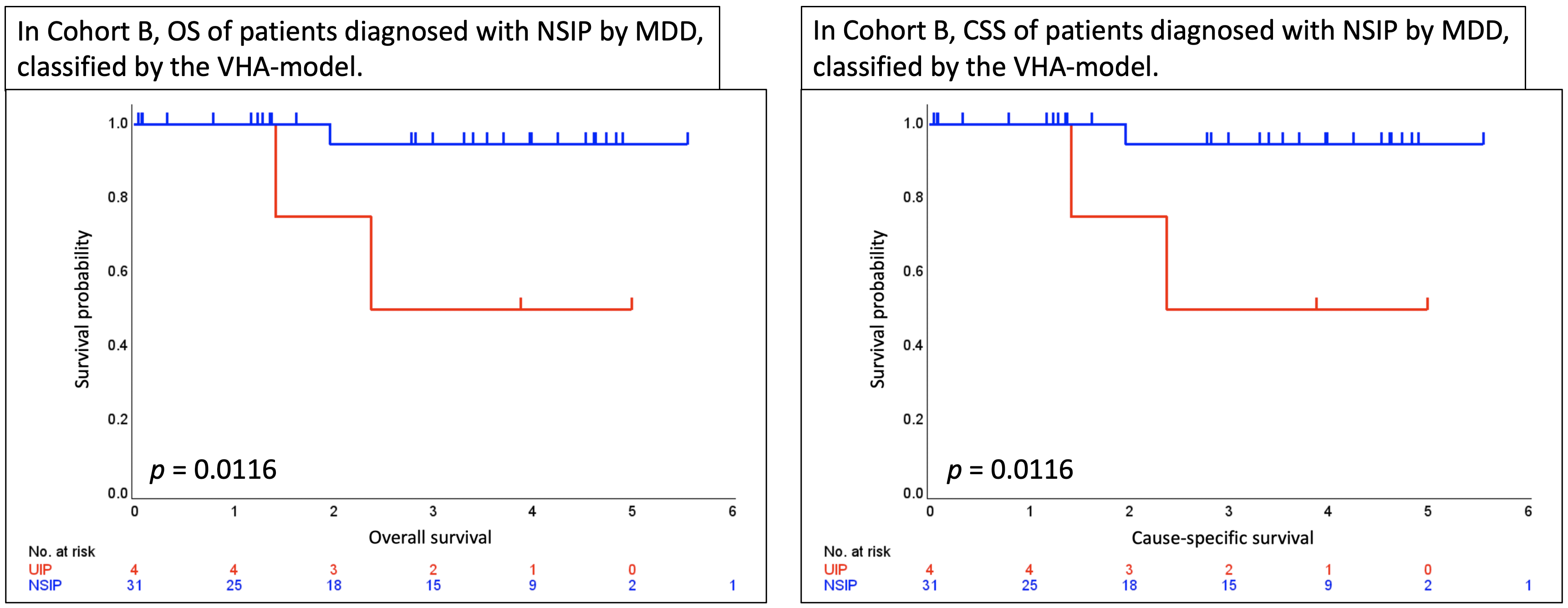


Figure S3B

Figure S3. Kaplan–Meier survival curves for OS and cause-specific survival (CSS) in Cohort B, comparing the VHA-model-based UIP vs. NSIP reclassification among patients originally diagnosed by MDD as IPF/UIP (left panels) or as NSIP (right panels). **(A)** Kaplan–Meier survival curves of OS and CSS in patients diagnosed with IPF/UIP by MDD. **(B)** Kaplan–Meier survival curves of OS and CSS in patients diagnosed with NSIP by MDD. In the MDD-diagnosed IPF/UIP and NSIP groups, patients classified as UIP by the VHA-model had significantly worse OS and CSS compared with those classified as NSIP by the VHA-model. The VHA-model refers to a model formulated based on the kurtosis, skewness, and entropy obtained from histogram analysis of each lung lobe.

OS overall survival, CSS cause-specific survival, MDD multidisciplinary discussion, IPF idiopathic pulmonary fibrosis, UIP usual interstitial pneumonia, NSIP nonspecific interstitial pneumonia.
